# Niclosamide Suppresses Migration and Invasion of Human Osteosarcoma Cells by Repressing TGFBI Expression via the ERK Signaling Pathway

**DOI:** 10.3390/ijms23010484

**Published:** 2022-01-01

**Authors:** Liang-Tsai Yeh, Chiao-Wen Lin, Ko-Hsiu Lu, Yi-Hsien Hsieh, Chao-Bin Yeh, Shun-Fa Yang, Jia-Sin Yang

**Affiliations:** 1 Department of Anesthesiology, Changhua Christian Hospital, Changhua 500, Taiwan; 68990@cch.org.tw; 2 School of Medicine, Chung Shan Medical University, Taichung 402, Taiwan; cshy307@csh.org.tw (K.-H.L.); sky5ff@gmail.com (C.-B.Y.); 3 College of Medicine, National Chung Hsing University, Taichung 402, Taiwan; 4 Institute of Oral Sciences, Chung Shan Medical University, Taichung 402, Taiwan; cwlin@csmu.edu.tw; 5 Department of Dentistry, Chung Shan Medical University Hospital, Taichung 402, Taiwan; 6 Department of Orthopedics, Chung Shan Medical University Hospital, Taichung 402, Taiwan; 7 Institute of Medicine, Chung Shan Medical University, Taichung 402, Taiwan; hyhsien@csmu.edu.tw; 8 Department of Medical Research, Chung Shan Medical University Hospital, Taichung 402, Taiwan; 9 Department of Emergency Medicine, Chung Shan Medical University Hospital, Taichung 402, Taiwan

**Keywords:** niclosamide, osteosarcoma, migration, TGFBI

## Abstract

Osteosarcoma is a highly common malignant bone tumor. Its highly metastatic properties are the leading cause of mortality for cancer. Niclosamide, a salicylanilide derivative, is an oral antihelminthic drug of known anticancer potential. However, the effect of niclosamide on osteosarcoma cell migration, invasion and the mechanisms underlying have not been fully clarified. Therefore, this study investigated niclosamide’s underlying pathways and antimetastatic effects on osteosarcoma. In this study, U2OS and HOS osteosarcoma cell lines were treated with niclosamide and then subjected to assays for determining cell migration ability. The results indicated that niclosamide, at concentrations of up to 200 nM, inhibited the migration and invasion of human osteosarcoma U2OS and HOS cells and repressed the transforming growth factor beta-induced protein (TGFBI) expression of U2OS cells, without cytotoxicity. After TGFBI knockdown occurred, cellular migration and invasion behaviors of U2OS cells were significantly reduced. Moreover, niclosamide significantly decreased the phosphorylation of ERK1/2 in U2OS cells and the combination treatment of the MEK inhibitor (U0126) and niclosamide resulted in the intensive inhibition of the TGFBI expression and the migratory ability in U2OS cells. Therefore, TGFBI derived from osteosarcoma cells via the ERK pathway contributed to cellular migration and invasion and niclosamide inhibited these processes. These findings indicate that niclosamide may be a powerful preventive agent against the development and metastasis of osteosarcoma.

## 1. Introduction

Osteosarcoma is a highly common type of malignant orthotopic bone tumor and mainly occurs in children and young adults. Tumors often develop in the metaphyseal growth plates of the long bones, originating in the mesenchymal tissue [1,2]. Current comprehensive treatments for patients with osteosarcoma typically include chemotherapy before and after surgery in combination with the surgical removal of the tumor [3]. The survival rate of patients with localized osteosarcoma has improved in recent years with advances in therapeutic approaches [4]. However, due to the strong invasiveness of osteosarcoma and its rapid progression, the prognosis for patients with metastatic osteosarcoma remains unfavorable [5,6]. Tumor metastasis and relapse remain the leading causes of mortality for patients with osteosarcoma [7,8]. Thus, identifying effective drugs for the treatment of osteosarcoma is crucial.

Niclosamide, a type of salicylanilide derivative, is a Food and Drug Administration–approved oral antihelminthic drug that has been used worldwide to treat millions of patients with tapeworm (cestodes) infections [9]. Niclosamide is included in the World Health Organization’s list of essential medicines [10], and is well tolerated [11] and safe [12,13] in humans. Niclosamide is a multifunctional drug that is systemically administered. It has been extensively studied for treating various diseases, including viral and microbial infections; metabolic diseases, such as Type II diabetes; nonalcoholic steatohepatitis and nonalcoholic fatty liver disease; artery constriction; endometriosis; neuropathic pain; rheumatoid arthritis; sclerodermatous-graft-versus-host disease; systemic sclerosis, Parkinson’s disease; and cancer [14,15]. Niclosamide has also been repeatedly demonstrated to exhibit effective anticancer activity against various cancers, including osteosarcoma [14,15]. Li et al. reported that niclosamide inhibits proliferation, and induces apoptosis and cell-cycle arrest in human osteosarcoma cells [16]. Liao et al. reported that niclosamide can effectively inhibit 143B and MG-63 osteosarcoma cell proliferation [17]. Specifically, niclosamide induces osteosarcoma cell apoptosis and G1 arrest and inhibits cell cycle progression by targeting multiple signaling pathways including, the transcription activity of E2F1, AP-1, and c-Myc. Niclosamide also inhibits tumor growth in a mouse xenograft model of human osteosarcoma cells [17]. 

The transforming growth factor beta induction (TGFBI) protein is a secreted protein localized to the extracellular matrix [18]. TGFBI plays important roles in regulating numerous biological functions and promoting tumor progression in established cancers [18,19]. A previous study demonstrated that TGFBI knockdown effectively inhibited the cell invasion and migration of osteosarcoma [20]. Guo et al. revealed that TGFBI was up-regulated in glioma cells and played a promoting role in the cell migration of glioma cells via AKT signaling pathway [21]. Moreover, Lee et al. demonstrated that TGFBI suppresses endothelial cells angiogenesis and migration by suppressing the activation of Src and ERK signaling pathways [22]. Although niclosamide has been considered to be a potential anticancer drug, the molecular mechanism underlying its inhibition of TGFBI expression and osteosarcoma metastasis has not been well delineated. This study aimed to investigate the role of niclosamide in human osteosarcoma cell migration and invasion.

## 2. Results

### 2.1. Cytotoxicity of Niclosamide on U2OS and HOS Osteosarcoma Cells

**Figure 1:**
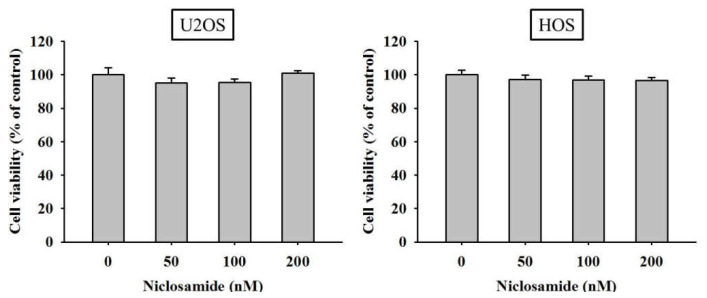
Effects of niclosamide on the cell viability of U2OS and HOS cells. The viability of U2OS and HOS cells treated with niclosamide (0–200 nM) for 24 h was detected through MTT assay, and the effects of niclosamide are illustrated after quantitative analysis. Results are shown as mean ± S.D. *n* = 6. One way ANOVA followed by Dunnett’s post hoc test was used. U2OS: F = 0.689, *p* = 0.571; HOS: F = 2.489, *p* = 0.090.

The cell viability of U2OS and HOS osteosarcoma cells at concentrations of 50, 100, and 200 nM niclosamide for 24 h was not significantly different from that of the controls (0 nM) in the MTT colorimetric assay (Figure 1). Thus, 24 h treatment with niclosamide at up to 200 nM had no cytotoxic effect on U2OS and HOS osteosarcoma cell lines. Thus, we used this concentration range for niclosamide in all subsequent experiments to investigate its antimetastatic properties.

### 2.2. Niclosamide Represses U2OS and HOS Cells Motility, Migration, and Invasion

**Figure 2:**
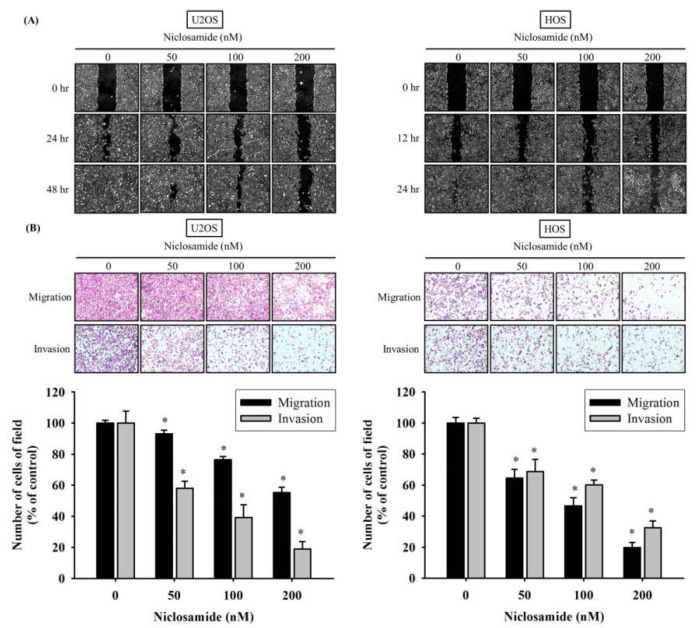
Effects of niclosamide on in vitro cellular motility, migration, and invasion of U2OS and HOS cells. (**A**) After different concentrations (0, 50, 100, and 200 nM) of niclosamide treatment for different time intervals in U2OS (0, 24, and 48 h) and HOS (0, 12, and 24 h) cells were detected by the wound-healing assay. (**B**) Cell migration and invasion assays after various concentrations (0, 50, 100, and 200 nM) of niclosamide treatment for 24 h in U2OS and HOS cells were measured, and illustrated after quantitative analysis. Results are shown as mean ± S.D with three independent experiments. * Significantly different: *p* < 0.05, when compared with the control group (0 nM).

To determine U2OS and HOS cells’ migratory and invasive abilities in vitro, wound healing assay and modified Boyden chamber were employed. After U2OS cells were treated for 24 h and 48 h and HOS cells for 12 h and 24 h, niclosamide significantly reduced the motility of U2OS and HOS cells in the wound healing assay (Figure 2A). In the assay of migration capacity, the niclosamide treated U2OS and HOS cells reduced to 7–45% and 36–80%, at applied concentration (Figure 2B). Regarding the invasion ability, similar results were observed and the strongest inhibitory effects were at 200 nM which suppressed the invasion rate to 82% and 68% in U2OS and HOS cells, respectively (Figure 2B). 

### 2.3. Niclosamide Decreases TGFBI Expression of U2OS Cells

**Figure 3:**
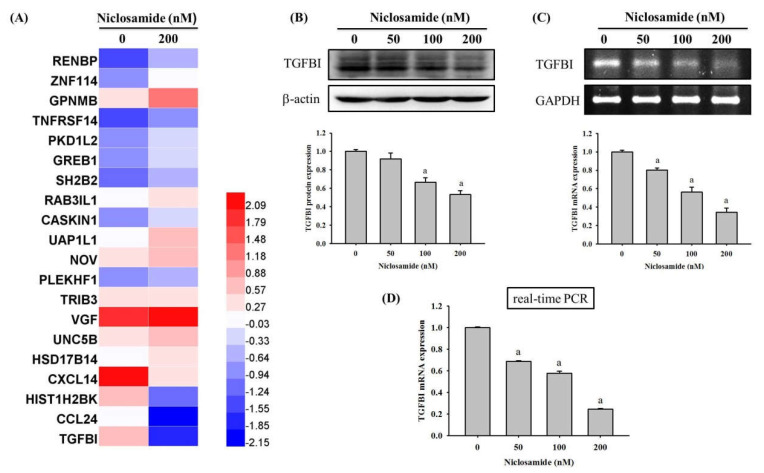
TGFBI expression of niclosamide-treated in osteosarcoma U2OS cells. (**A**) Heat map of the hierarchical clustering of 20 differentially expressed genes identified by RNA-Seq after treatment with 200 nM of niclosamide for 24 h in U2OS cells. (**B**) Western blot analysis, (**C**) RT-PCR, and (**D**) real-time PCR after various concentrations (0, 50, 100, and 200 nM) of niclosamide treatment for 24 h in U2OS cells were measured as described in the Materials and Methods section and the effects were illustrated after quantitative analysis. Results are shown as mean ±S.D with three independent experiments. One way ANOVA followed by Dunnett’s post hoc test was used. ^a^ Significantly different: *p* < 0.001, when compared with the control group (0 nM). Western blotting: F = 66.731, *p* < 0.001; RT-PCR: F = 159.980, *p* < 0.001; real-time PCR: F = 2237.390, *p* < 0.001.

To explore the downstream regulated protein of niclosamide, RNA sequencing (RNA-Seq) technology was conducted. As shown in Figure 3A, the results indicated that TGFBI was the most significantly downregulated gene in U2OS cells after treatment with 200 nM niclosamide for 24 h (Figure 3A). We subsequently performed Western blot analysis, reverse transcription-polymerase chain reaction (RT-PCR), and real-time PCR to validate the findings of the RNA-Seq. Our results revealed that niclosamide reduced the TGFBI protein and mRNA levels by 47% and 66% in U2OS cells at the highest concentration (200 nM) (Figure 3B,C). Moreover, real time PCR assay showed that a 24-h treatment with 100 nM of niclosamide showed a 42% reduction, while a 24-h treatment with 200 nM of niclosamide decreased 75% TGFBI mRNA level in U2OS cells (Figure 3D).

### 2.4. TGFBI Knockdown Reduces Migration and Invasion of U2OS Cells

**Figure 4:**
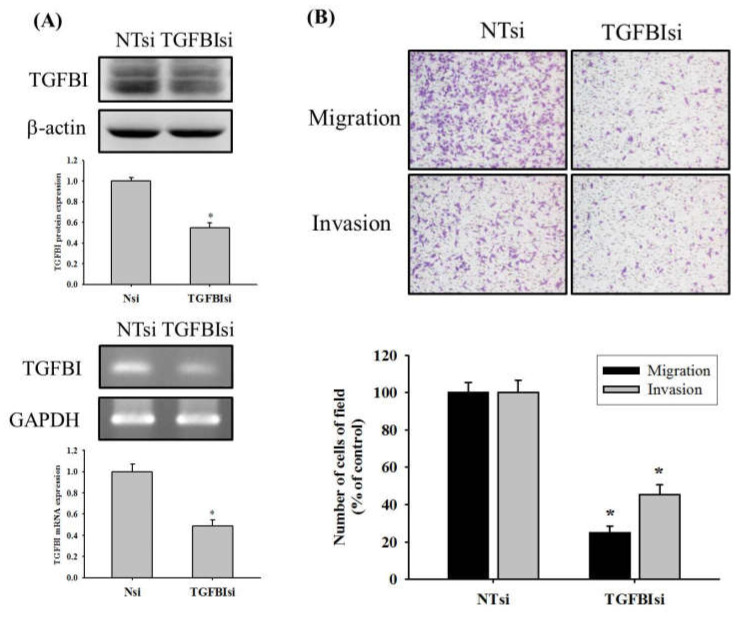
Effects of TGFBI knockdown on cell migration and invasion of U2OS cells. (**A**) Using western blot analysis, and RT-PCR to confirm siRNA directly against TGFBI expression for 72 h. (**B**) Modified Boyden chamber assays with or without Matrigel coating after treatment of TGFBI siRNA for 24 h in U2OS cells were conducted and the effects were illustrated after quantitative analysis. Results are shown as mean ± S.D with three independent experiments. Student’s *t*-test was used. * Significantly different: *p* < 0.05, when compared with the control group (NTsi). NTsi: non-target small interfering RNA; TGFBIsi: TGFBI small interfering RNA.

To further verify whether the reduction of TGFBI influences the migratory and invasive properties of U2OS cells, we transfected U2OS cells with a small interfering RNA (siRNA) targeting TGFBI expression for 72 h. We confirmed knockdown of TGFBI protein and mRNA levels through Western blotting and RT-PCR in U2OS cells, respectively (Figure 4A). Our results revealed that TGFBI siRNA reduced the TGFBI protein and mRNA levels by 45% and 54% in U2OS cells (Figure 4A). We then performed Boyden chamber migration and modified Matrigel invasion assays for 24 h while using siRNA of TGFBI to compare the amount of migratory and invasive cells. As shown in Figure 4B, the knockdown of TGFBI significantly decreased the migration and invasion by 75% and 54% of U2OS cells (*p* < 0.05) (Figure 4B).

### 2.5. Niclosamide Inhibits the ERK Signaling Pathway in U2OS Cells

**Figure 5:**
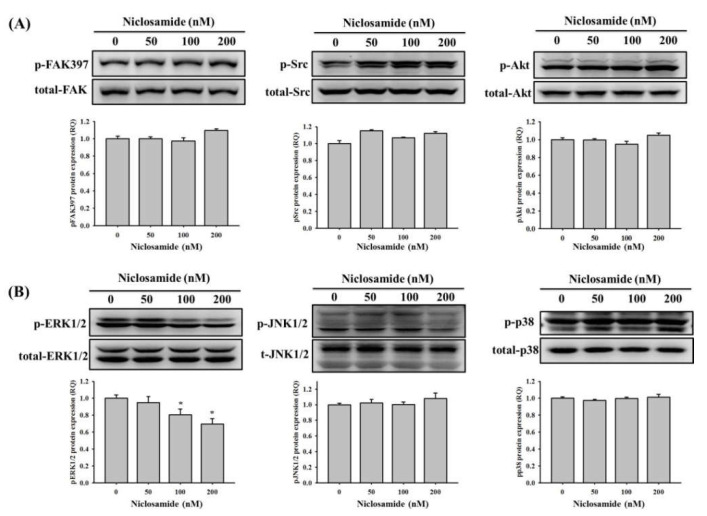
Effects of niclosamide on FAK-Src, PI3K-Akt and MAPKs signaling pathway in U2OS cells. Western blot analysis for total or phosphorylated forms of (**A**) FAK, Src, Akt, (**B**) ERK 1/2, JNK 1/2, and p38 after various concentrations (0, 50, 100, and 200 nM) of niclosamide treatment for 24 h in U2OS cells were measured as described in the Materials and Methods section. Results are shown as mean ± S.D with three independent experiments. One way ANOVA followed by Dunnett’s post hoc test was used. * *p* < 0.05, when compared with the control group (0 nM). P-ERK1/2: F = 15.004, *p* = 0.001.

**Figure 6:**
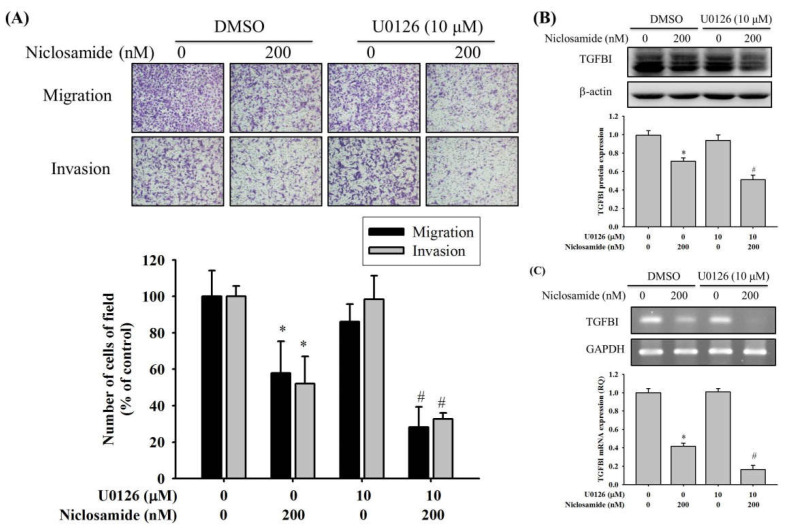
Effects of niclosamide on ERK to modulate TGFBI and biological behaviors of migration and invasion in U2OS cells. (**A**) Cell migration and invasion assays for effects of the inhibitor of MEK (U0126) for 24 h on U2OS cells with or without treatment of 200 nM niclosamide were measured and subsequently subjected to quantitative analysis. (**B**) Western blot analysis and (**C**) RT-PCR for U2OS cells with or without treatment of 200 nM niclosamide to determine effects of U0126 on TGFBI expressions were conducted. Results are shown as mean ± S.D with three independent experiments. * Significantly different, *p* < 0.05, when compared with U2OS cells without treatment of niclosamide and U0126. # Significantly different, *p* < 0.05, when compared with U2OS cells with niclosamide but without U0126.

Since phosphatidylinositide-3 kinase-Akt (PI3K-Akt), and mitogen-activated protein kinase (MAPK) pathways may be downstream signaling of TGFBI [21,23], Western blot analysis was employed to further investigate its molecular mechanisms. In the analysis, FAK-Src, PI3K-Akt, and MAPK signaling pathways were detected in U2OS cells after treatment with niclosamide for 24 h. Consequently, niclosamide decreased the phosphorylation of ERK1/2 by 31% at the concentration of 200 nM in U2OS cells but exerted no obvious effect on FAK, Src, Akt, JNK 1/2, or p38, including on their phosphorylation (Figure 5A,B). To recognize whether the suppression of ERK 1/2 phosphorylation by niclosamide interferes with the actions of TGFBI expression and cell migration in U2OS cells, an inhibitor of MEK pathway (U0126) was used. We performed Boyden chamber migration and modified Matrigel invasion assays to compare the number of migratory and invasive cells. As expected, the cell migration and invasion ability was suppressed when the cells were treated with niclosamide only. The combination treatment of U0126 (10 μM) and niclosamide (200 nM) resulted in the intensive inhibition of the migration and invasion ability by 72% and 67% in U2OS cells, respectively (Figure 6A). Moreover, the combination treatment of U0126 (10 μM) and niclosamide (200 nM) also resulted in the intensive inhibition of TGFBI protein and mRNA expression by 49% and 83% in U2OS cells (Figure 6B,C). Overall, these results indicate that the ERK1/2 pathway plays a key upstream role in U2OS cells treated with niclosamide and modulates cell migration and invasion.

## 3. Discussion

In the present study, we revealed that niclosamide can effectively inhibit the cell migration and invasion of osteosarcoma cell lines by reducing the expression of TGFBI. Guo et al. demonstrated that TGFBI could promote osteosarcoma cells metastasis by interacting with integrin α2β1 while knockdown of TGFBI inhibited the cell migration ability [20]. Similarly, our results demonstrated that niclosamide inhibited the migration and invasion of human osteosarcoma cells via repressing the TGFBI expression. These results indicate that TGFBI may act as a major contributor to the metastatic potential of osteosarcoma and function as a potential therapeutic target for the treatment of osteosarcoma.

Mounting evidence indicates that niclosamide is a multifunctional drug that can regulate multiple intracellular signaling pathways in various cancers [14]. The antimetastatic activity of niclosamide is associated with inhibitory effects on several signaling pathways, such as the STAT3 pathway in melanoma, [24] in oral squamous cell carcinoma, [25] and in prostate cancer [26]; Wnt/β-catenin signaling in oral squamous cell carcinoma [27] and in adrenocortical carcinoma [28]; Notch signaling in colon cancer [29]; and IGF signaling in ovarian cancer [30]. Our study demonstrated that niclosamide suppresses the ERK pathway to inhibit osteosarcoma cell migration and invasion. 

The ERK signaling is a pathway related to other different signaling cascades and, therefore, to various biological processes in various cancers [31,32,33]. In osteosarcoma, Chen et al, reported that c-Met Inhibitor suppresses osteosarcoma progression via the ERK1/2 pathway [34]. In addition, Liu et al demonstrated that monocyte chemoattractant protein-1 regulates cell mobility via ERK/AP-1 pathway in osteosarcoma cells [35]. However, inhibiting ERK pathway could imply that other processes may be repressed. Interestingly, studies have revealed that niclosamide treatment leads to the downregulation of MAPK/ERK, and STAT3 prosurvival signal transduction pathways to further reduce human glioblastoma U-87 MG cell viability [36]. Niclosamide inhibits the phosphorylation of the ERK, Mnk1, and eIF4E signaling pathways, and it also inhibits the proliferation of and induces apoptosis in chronic myeloid leukemia cells [37]. However, whether the presence of other biological processes that might be affected by niclosemide should be investigated in the future.

## 4. Materials and Methods

### 4.1. Cell Culture

Cells were obtained from American Type Culture Collection (Manassas, VA, USA), and comprised human osteosarcoma U2OS (15-yr-old female individual; Epithelial cells) (ATCC: Cat# HTB-96) and HOS (13-yr-old female individual; mixed, fibroblast and epithelial like cells) (ATCC: Cat# CRL-1543) cell lines. The U2OS and HOS cells were supplemented with 10% FBS and 1% penicillin and streptomycin and cultured in Dulbecco’s modified Eagle’s medium and minimal essential medium, respectively. The cell lines were maintained at 37 °C in humidified atmosphere of a 5% CO_2_ incubator.

### 4.2. Cell Viability Assay

For the cell viability experiment, we plated 8.5 × 10^4^/well U2OS cells and 8 × 10^4^/well HOS cells in 24-well plates for 24 h and then treated the plates with different concentrations (0, 50, 100, and 200 nM) of niclosamide at 37 °C for 24 h. After the exposure period, a MTT (3-(4,5-dimethylthiazol-2-yl)-2,5-diphenyltetrazolium bromide) colorimetric assay was performed to determine the cytotoxicity of niclosamide. The medium was changed, and the cells were incubated with MTT (0.5 mg/mL), diluted with medium for 4 h, and then removed. The viable cells produced formazan that was directly proportional to cell viability. Formazan is soluble in isopropanol and was measured spectrophotometrically at 563 nm.

### 4.3. Wound Healing Assay

To examine whether niclosamide alters the motility of U2OS and HOS cells, we plated 2 × 10^6^ U2OS cells and 1.5 × 10^6^ HOS cells on 6 cm dishes for 24 h and wounded them by scratching the dishes with a pipette tip. Subsequently, the cells were incubated in a medium containing 0.5% FBS and niclosamide for 0, 24, and 48 h for U2OS cells and for 0, 12, and 24 h for HOS cells. Cells were photographed using a phase-contrast microscope (100×).

### 4.4. Cell Migration and Invasion Assays

Cells were seeded into the upper section of the Boyden chamber (Neuro Probe, Cabin John, MD, USA) and covered with 8-μm-pore polycarbonate membrane filters with or without Matrigel in the amounts of 1.0 × 10^4^ for the U2OS cells and 7.5 × 10^3^ for the HOS cells. Cells were then incubated at 37 °C in a 5% CO_2_ incubator for 24 h. The cells that had migrated (without coating Matrigel) and invaded (coating with Matrigel) the lower surface of the membrane were fixed with methanol for 10 min and stained with Giemsa for 4 h; nonmigrating and noninvading cells on the upper surface were wiped off gently with a cotton swab. Finally, the random fields were counted under a light microscope.

### 4.5. Protein Extraction and Western Blot Analysis

Cells were washed with PBS twice and added to a PRO-PREP protein extraction kit (iNtRON Biotechnology Co., Seoul, Korea) before being scraped. Total cell lysates were subjected to a centrifugation of 13,200 rpm for 30 min at 4 °C; subsequently, the supernatant was collected and the protein concentration was measured by a Bradford protein assay. Cell lysates (20 μg) were separated in 10%–12.5% SDS-PAGE before being transferred onto nitrocellulose (NC) paper. Subsequently, NC paper was blocked with 5% bovine serum albumin in Tris-buffered saline Tween-20 (TBST) for 1 h, followed by probing overnight at 4 °C with the primary antibody. The primary antibodies were used as follows: TGFBI (ab170874, abcam), β-actin (ab8226, abcam), FAK (610088, BD Biosciences), phospho-FAK (611807, BD Biosciences), Src (ADI-905-678, Enzo Life Sciences), phospho-Src (2101, Cell Signaling Technology), Akt (610860, BD Biosciences), phospho-Akt (4060, Cell Signaling Technology), Erk1/2 (9102, Cell Signaling Technology), phospho-Erk1/2 (4370, Cell Signaling Technology), JNK1/2 (9258, Cell Signaling Technology), phospho-JNK1/2 (9251, Cell Signaling Technology), p38 (612169, BD Biosciences), phospho-p38 (612281, BD Biosciences). After washing with TBST, the NC paper was incubated for 2 h at room temperature with horseradish peroxidase (HRP)-conjugated secondary antibodies. The intensity of each band was visualized using enhanced chemiluminescence (ECL) solution (Millipore, Billerica, MA, USA) and detected by luminometer.

### 4.6. RNA Extraction, Reverse Transcription-Polymerase Chain Reaction (RT-PCR), and Real-Time PCR

Total RNA was extracted using a Total RNA Mini Kit (Geneaid, New Taipei City, Taiwan) and reverse transcribed into complementary DNA (cDNA) using a High Capacity cDNA Reverse Transcription Kit (Applied Biosystems, Thermo Fisher Scientific, Chino, CA, USA) according to the manufacturer’s instructions. The final products were separated by electrophoresis in 3% agarose gel and detected with ethidium bromide staining. GAPDH was used as internal control. A real-time PCR assay was performed by using Fast SYBR Green Master Mix (Applied Biosystems) and appropriate primers on a StepOnePlus Real-Time PCR System (Applied Biosystems). The PCR primers and PCR cycle program were listed in the Supplementary Table S1. The cycle threshold value was used as an indicator of the amount of target RNA, and gene expression was normalized to GAPDH and quantified according to the 2−ΔΔCt method.

### 4.7. Small Interfering RNA (siRNA) and Transient Transfection

For the silencing of the transforming growth factor beta-induced protein (TGFBI) expression, a unique siRNA inhibiting human TGFBI (s14070) and negative-control siRNA (4390844) were purchased from Applied Biosystems. We plated 8 × 10^5^ U2OS cells on 6-cm dishes for 24 h and transfected with 150 pmol TGFBI siRNA and the negative-control siRNA for 72 h using lipofectamine RNAiMAX reagent (Invitrogen, Carlsbad, CA, USA).

### 4.8. Statistical Analysis

The experimental data are expressed as means ± standard deviations, and the differences between the groups were analyzed using Student’s *t*-test. For more than two groups, the data was performed by a one way ANOVA followed by Dunnett’s post hoc tests. SPSS software was used for statistical analysis, and *p* < 0.05 indicated statistical significance.

## 5. Conclusions

In conclusion, our study is first to demonstrate that niclosamide suppresses the migration and invasion of human osteosarcoma U2OS and HOS cells by inhibiting TGFBI expression via the ERK signaling pathway. These results demonstrate that niclosamide possesses antimetastatic properties in human osteosarcoma cells and can be developed into a potential chemotherapeutic agent for osteosarcoma.

## Data Availability

The data presented in this study are available on request from the corresponding author.

## Supplementary Materials

The following supporting information can be downloaded at: https://www.mdpi.com/article/10.3390/ijms23010484/s1.
