# Supplementary material for: Niclosamide Suppresses Migration and Invasion of Human Osteosarcoma Cells by Repressing TGFBI Expression via the ERK Signaling Pathway
Source: Int J Mol Sci. 2022 Jan 1;23(1):484. doi: 10.3390/ijms23010484 (PMC8745393; doi:10.3390/ijms23010484)
Supplement: Supplementary file 1 [file ijms-23-00484-s001.zip › ijms-1522921-supplementary.pdf]

Supplementary Table S1: Primers used for RT-PCR and real-time PCR.

Primer	Sequence 5'→3'	Annealing Temperature	Cycle	Size (bp)
TGFBI (RT-PCR; real-time PCR)	Forward: CGTAATAGCCTCTGCATTGAGAAC<br>Reverse: CAGCATGCTAAAGCGATTGTCT	62℃	30	150
GAPDH (RT-PCR)	Forward: CGGAGTCAACGGATTTGGTCGTAT<br>Reverse: AGCCTTCTCCATGGTGGTGAAGAC	65℃	16	307
GAPDH (real-time PCR)	Forward: GGAGCGAGATCCCTCCAAAAT<br>Reverse: GGCTGTTGTCATACTTCTCATGG	-	-	197
